# The Half-Yearly Compendium

**Published:** 1869-06

**Authors:** 


The Half-Yearly Compendium.—The number of this periodical for January has now appeared. It is full of the choicest selections on all branches of medical science, and should be in the hands of every physician. No synopsis of professional works, or articles, at all compare with it, either in the diversity of sources consulted, or the number of valuable facts accumulated.

The annoying delay in the publication of this number will be avoided in future. The next number may be confidently looked for by July 10th.—*Medical and Surgical Reporter.*

